# From failure to fate: Pressure-driven exhaustion in solid-tumor CAR T-cell therapy

**DOI:** 10.1016/j.cellin.2026.100377

**Published:** 2026-10-01

**Authors:** Pei Wang, Yuehong Zhou, Yuan Wang, Yangyang Li, Xue Xiao, Chenhui Wang

**Affiliations:** a Department of Pathology, Sichuan Provincial People's Hospital, School of Medicine, University of Electronic Science and Technology of China, Chengdu, Sichuan, 610072, China; b The Key Laboratory for Human Disease Gene Study of Sichuan Province and the Department of Laboratory Medicine, Sichuan Provincial People's Hospital, School of Medicine, University of Electronic Science and Technology of China, Chengdu, Sichuan, 610072, China; c Research Unit for Blindness Prevention of the Chinese Academy of Medical Sciences, Sichuan Academy of Medical Sciences and Sichuan Provincial People's Hospital, Chengdu, Sichuan, 610072, China; d School of Medicine, University of Electronic Science and Technology of China, Chengdu, Sichuan, 610054, China; e Personalized Drug Research and Therapy Key Laboratory of Sichuan Province, Sichuan Provincial People's Hospital, Chengdu, Sichuan, 610072, China; f Chuan-Yu Joint Key Laboratory for Pathological and Laboratory Medicine, Jinfeng Laboratory, Chongqing, 400039, China

**Keywords:** CAR T-cell therapy, Solid tumor, T cell exhaustion, CAR T-cell fate, Tumor microenvironment

## Abstract

Chimeric antigen receptor (CAR) T-cell therapy has achieved remarkable and durable responses in hematologic malignancies, but its efficacy in solid tumors remains limited and frequently transient. In this Review, we propose that interacting tumor barriers divert persistently engaged CAR-T cells toward dysfunctional and exhausted states. Defective trafficking, antigen heterogeneity, incomplete tumor clearance, metabolic deprivation, stromal exclusion, immunosuppressive cellular networks, inhibitory cytokines, chronic CAR signaling, and manufacturing-imprinted stress collectively shape the stimulation history and functional trajectory of CAR T-cells. Mechanistically, these pressures converge on NFAT–AP-1 imbalance, TOX/TOX2 and NR4A activation, mitochondrial dysfunction, and exhaustion-associated epigenetic remodeling. This process creates a clinically relevant continuum between TCF1-positive progenitor-exhausted cells that retain self-renewal and checkpoint-responsive proliferative potential, and terminally exhausted cells with limited regenerative capacity. Building on this failure–pressure–fate model, we propose a mechanism-informed therapy framework that relates dominant failure mechanisms and residual CAR T-cell plasticity to potential intervention priorities. This approach may help match combination strategies to the dominant tumor pressure and CAR T-cell state, including stromal remodeling, metabolic engineering, cytokine armoring, checkpoint modulation, epigenetic intervention, and remanufacturing. Overall, this Review provides a fate-based framework for designing next-generation CAR T-cell therapies capable of achieving durable control in solid tumors.

**Table:** Abbreviations

Abbreviation	Definition
AP-1	Activator protein 1
ATAC	Assay for transposase-accessible chromatin
CAF	Cancer-associated fibroblast
CAR	Chimeric antigen receptor
ECM	Extracellular matrix
MDSC	Myeloid-derived suppressor cell
NFAT	Nuclear factor of activated T cells
ROS	Reactive oxygen species
scFv	Single-chain variable fragment
TAM	Tumor-associated macrophage
Tcm	Central memory T cells
terminal Tex	Terminally exhausted T cells with limited proliferative and regenerative potential
Tex	Exhausted T cells; a heterogeneous population spanning progenitor, intermediate and terminal states
TME	Tumor microenvironment
Tn	Naïve T cells
Tpex	Progenitor exhausted T cells with self-renewal capacity and the ability to generate more differentiated progeny
Treg	Regulatory T cells
Tscm	Stem cell memory T cells

## 1 Introduction

Chimeric antigen receptor (CAR) T-cell therapy has transformed cancer immunotherapy by enabling genetically engineered T cells to mediate potent and durable antitumor responses. Its clinical success has been most evident in hematologic malignancies, where CD19-targeted CAR T-cells induce high remission rates in relapsed or refractory B-cell acute lymphoblastic leukemia (Maude et al., 2018) and diffuse large B-cell lymphoma (Neelapu et al., 2017), while BCMA-directed CAR T-cell therapies achieve deep and sustained responses in multiple myeloma (Berdeja et al., 2021). These outcomes underscore the importance of sustained CAR T-cell persistence and functional integrity for long-term disease control.

In contrast, the clinical efficacy of CAR T-cell therapy in solid tumors has been limited and heterogeneous (Haas et al., 2019). Although objective responses have been observed in glioblastoma and neuroblastoma, durable remission remains restricted to a subset of patients (Brown et al., 2016; Del Bufalo et al., 2023; Li et al., 2025). Notably, recent advances have begun to shift this landscape. In June 2026, China's National Medical Products Administration (NMPA) approved satricabtagene autoleucel for CLDN18.2-positive, HER2-negative advanced gastric/gastroesophageal junction adenocarcinoma after at least two prior lines of therapy, marking the first global approval of a CAR T-cell therapy for a solid tumor. Despite these advances, primary resistance, incomplete tumor eradication, limited persistence, and relapse remain major challenges.

The limited efficacy of CAR T-cell therapy in solid tumors reflects a complex interplay of tumor-intrinsic and microenvironmental barriers. Physical constraints such as abnormal vasculature and a dense extracellular matrix (ECM) restrict T-cell infiltration (Caruana et al., 2015; Deng et al., 2020), while metabolic stressors including hypoxia, nutrient deprivation, and acidosis impair cellular fitness (Feng et al., 2026; Hatae et al., 2024; Hu et al., 2025). In parallel, immunosuppressive components such as regulatory T cells, myeloid-derived suppressor cells (MDSCs), tumor-associated macrophages (TAMs), and inhibitory cytokines further dampen CAR T-cell activity (Burga et al., 2015; Rodriguez-Garcia et al., 2021; Tang et al., 2020). Antigen heterogeneity promotes immune escape, and low-level antigen expression in normal tissues raises safety concerns (Morgan et al., 2010; O'Rourke et al., 2017). These external pressures interact with CAR-intrinsic factors, including receptor design, tonic signaling, and differentiation state, collectively shaping therapeutic outcomes. Importantly, diminished CAR T-cell function does not uniformly reflect exhaustion but may arise from multiple dysfunctional states. As summarized in Table 1, exhaustion, senescence, anergy, and NK-like dysfunctional states can share impaired proliferation or effector function but differ in their dominant triggers, molecular features, and reversibility. Distinguishing these states is therefore important when interpreting CAR T-cell failure and considering potential rescue strategies.

**Table 1:** Distinguishing exhaustion from other dysfunctional states in CAR T-cells.

State (schematic)	Main trigger	Core features	Reversibility/relation to exhaustion	Reference
	Persistent antigen exposure and chronic CAR signaling	Progressive loss of proliferation and effector function; TOX/NR4A-associated programs; epigenetic remodeling	Stage-dependent; progenitor-like cells retain greater plasticity, whereas terminally exhausted cells are poorly reversible	(Ahn et al., 2024; Chow et al., 2022; Good et al., 2021)
	Replicative stress, prolonged expansion, DNA damage	Stable cell-cycle arrest; reduced proliferative capacity; p16/p21 and DNA-damage-associated features	Usually difficult to reverse once stable arrest is established; distinguished from exhaustion by persistent cell-cycle arrest	(Pérez-Criado et al., 2026; Tao et al., 2024; Titov et al., 2022)
	Antigen recognition with insufficient costimulation or dominant inhibitory signaling	Poor IL-2 production and proliferation despite receptor engagement	Can be reversible when productive signaling is restored; reflects failed activation rather than progressive exhaustion	(Jenkins et al., 2023; Titov et al., 2022)
	Chronic antigen exposure and CAR stimulation	Acquisition of NK-associated transcriptional and receptor features with loss of T-cell function	Overlaps with exhaustion and may represent a chronic-stimulation-associated dysfunctional trajectory	Good et al. (2021)

Exhaustion is a differentiation program associated with persistent antigen stimulation, characterized by progressive functional impairment and distinct transcriptional and epigenetic states (Gennert et al., 2021; Philip et al., 2017; Scharping et al., 2021). Exhausted T cells (Tex) comprise heterogeneous populations with different degrees of proliferative capacity and developmental plasticity. Progenitor exhausted T cells (Tpex), typically associated with TCF1 expression, retain self-renewal and the capacity to generate more differentiated progeny, whereas terminally exhausted T cells (terminal Tex) have limited regenerative potential. This distinction is particularly relevant to CAR T-cell therapy, although the extent to which engineered CAR T-cells recapitulate the differentiation hierarchy established in endogenous antigen-specific T cells remains incompletely defined. We therefore use the term Tpex-like CAR T-cells to describe CAR T-cell populations with progenitor exhaustion-associated features without assuming an identical lineage identity. Throughout this Review, exhaustion is considered a major pressure-driven fate trajectory of CAR T-cell dysfunction rather than an umbrella term encompassing all dysfunctional states. We provide a unified framework for understanding how clinical failure routes generate persistent CAR T-cell engagement, how pre-infusion susceptibility (Fig. 1) and post-infusion tumor-derived pressures shape subsequent CAR T-cell fate, and how these conditions remodel signaling, transcriptional, metabolic, and epigenetic programs. We further summarize emerging strategies to prevent exhaustion, restore function, or circumvent other dysfunctional states.

**Fig. 1:**
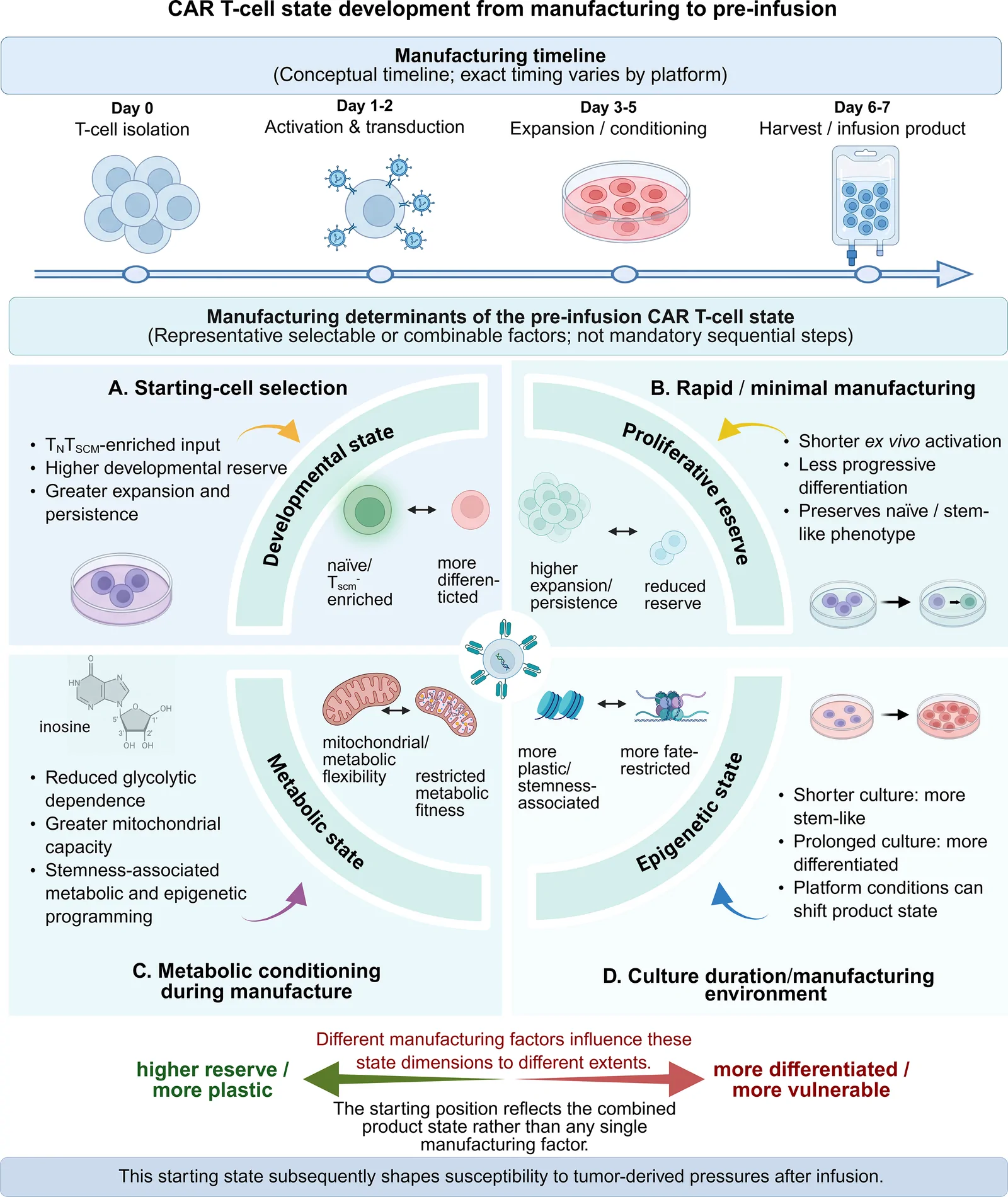
**Manufacturing determinants of the pre-infusion CAR T-cell state.** CAR T-cell manufacturing does not constitute a tumor-derived pressure but establishes the cellular starting state from which subsequent antigenic and microenvironmental pressures are interpreted. The upper timeline summarizes a conceptual manufacturing window from T-cell isolation, activation and transduction, through expansion or conditioning, to harvest of the infusion product; exact timing varies across manufacturing platforms. Representative selectable or combinable manufacturing determinants include starting-cell selection, rapid or minimally expanded manufacturing, metabolic conditioning, and culture duration/manufacturing environment. These factors do not act through a single pathway or as mandatory sequential steps. Instead, they differentially shape four major dimensions of the pre-infusion product state: developmental state, proliferative reserve, metabolic fitness, and epigenetic plasticity. Enrichment of naïve or stem-memory T cells and shortened ex vivo expansion favor a less differentiated product with greater proliferative reserve, whereas metabolic conditioning can enhance mitochondrial fitness and promote stemness-associated metabolic and epigenetic programs. Prolonged culture or other manufacturing conditions can shift the product toward more differentiated and less plastic states. The resulting infusion product therefore occupies a position along a continuum from higher-reserve, more plastic states to more differentiated and more vulnerable states. This pre-infusion starting state subsequently shapes the susceptibility of CAR T-cells to tumor-derived pressures after infusion.

## 2 Clinical activity is evident, but durable control remains limited

### 2.1 Demonstrated clinical activity highlights potential but not sustained benefit

CAR T-cell therapy has progressed from feasibility to demonstrable activity in solid tumors. In CLDN18.2-positive gastric or gastroesophageal junction cancer, CT041-ST-01 showed that satricabtagene autoleucel (satri-cel) improved median progression-free survival (PFS) from 1.77 to 3.25 months and increased the confirmed objective response rate (ORR) from 4% to 22% versus physician's choice (Qi et al., 2025). This provided randomized evidence of benefit in an epithelial malignancy, although the short PFS showed that tumor regression does not necessarily yield sustained control. In relapsed or refractory high-risk neuroblastoma, GD2-CART01 induced responses in 17 of 27 patients, including nine complete responses, and remained detectable for up to 30 months (Del Bufalo et al., 2023), indicating that durable benefit is possible when target expression, expansion, and persistence align.

### 2.2 Early tumor regression contrasts with limited durability in glioblastoma and other solid tumors

Glioblastoma provides a representative example of the discrepancy between initial antitumor activity and sustained clinical efficacy. Early-phase studies have demonstrated that CARv3-TEAM-E cells can induce rapid radiographic tumor regression; however, these responses were transient in the majority of treated patients (Choi et al., 2024). Similarly, bivalent EGFR806/IL-13Rα2 CAR T-cells have been shown to mediate tumor shrinkage, although confirmed objective responses remain infrequent and are often limited by treatment-associated inflammation, corticosteroid use, and ongoing disease progression (Bagley et al., 2025). Locoregional administration of IL-13Rα2-targeted CAR T-cells has achieved disease stabilization or transient regression in a subset of patients, yet durable responses are uncommon (Brown et al., 2024). In contrast, the BrainChild02 study reported no objective responses in pediatric patients with EGFR-positive central nervous system tumors (Gust et al., 2025). Collectively, these findings suggest that while advances in delivery strategies and multi-antigen targeting can enhance intratumoral activity, they do not consistently translate into durable clinical benefit.

Consistent with these observations, similar patterns have been reported across CAR T-cell programs targeting mesothelin, HER2, EGFR, CD133, PSMA, PSCA, B7-H3, GD2, CD70, and DLL3, as summarized in Table 2. Across these studies, clinical outcomes are predominantly characterized by stable disease, transient tumor regression, or isolated and unconfirmed responses, whereas reproducible and durable remissions remain rare. Notably, even in cases where CAR T-cell expansion, tumor infiltration, or occasional complete responses have been documented, multiple factors—including limited in vivo persistence, antigen heterogeneity, adaptive immunosuppressive mechanisms, treatment-related toxicity, and subsequent disease progression—continue to constrain long-term therapeutic efficacy.

**Table 2:** Clinical efficacy and principal limitations of CAR T-cell trials in solid tumors.

Trial identifier	Target	Tumor type(s)	Highest phase	Main failure or limitation	Reference
NCT02541370	CD133	Advanced hepatocellular carcinoma (phase II); pancreatic and colorectal cancers (phase I)	Phase II	Objective responses were uncommon and disease control was predominantly stable or transient; durable tumor eradication was not demonstrated, while transient cytopenias and hyperbilirubinemia were notable treatment-related toxicities.	(Dai et al., 2020; Wang et al., 2018)
NCT01935843	HER2	Advanced biliary tract and pancreatic cancers	Phase I	Only one partial response was observed and stable disease predominated, with a median PFS of 4.8 months; durable tumor control remained limited despite measurable CAR T-cell expansion and persistence.	Feng et al. (2018)
NCT01869166	EGFR	EGFR-positive advanced biliary tract cancer	Phase I	One complete response and 10 cases of stable disease were observed among 17 evaluable patients, but median PFS was only 4 months; most patients therefore lacked durable objective tumor regression.	Guo et al. (2018)
NCT03726515	EGFRvIII	Newly diagnosed, MGMT-unmethylated, EGFRvIII-positive glioblastoma	Phase I	CAR T-cell therapy combined with pembrolizumab showed no clear clinical efficacy, with a median PFS of 5.2 months; relapse tumors showed increased exhausted, regulatory and IFN-stimulated T-cell programs, indicating persistent adaptive immune suppression.	Bagley et al. (2024)
NCT05168423	EGFR806 + IL-13Rα2	Recurrent EGFR-amplified glioblastoma	Phase I	Tumor regression occurred in 62% of patients with measurable disease, but confirmed ORR was only 8% and median PFS was 1.9 months; grade 3 neurotoxicity and limited efficacy of retreatment constrained overall benefit.	Bagley et al. (2025)
NCT05660369	EGFRvIII + EGFR	Recurrent glioblastoma	Phase I	Rapid radiographic regression occurred after a single infusion, but responses were transient in two of three patients; CAR T-cell persistence declined within weeks, limiting durability of tumor control.	Choi et al. (2024)
NCT02208362	IL-13Rα2	Recurrent high-grade glioma or glioblastoma	Phase I	Disease stabilization or regression occurred in a subset of patients, but objective responses were uncommon and durable benefit was limited; determinants of response remained heterogeneous and were not prospectively validated.	Brown et al. (2024)
NCT02159716	Mesothelin	Pancreatic ductal adenocarcinoma, malignant pleural mesothelioma, and ovarian cancer	Phase I	The best overall response was stable disease in 11 of 15 patients, with no objective responses; CAR T-cells expanded transiently but showed poor persistence beyond 28 days and low intratumoral abundance.	Haas et al. (2019)
NCT04249947	PSMA	Metastatic castration-resistant prostate cancer	Phase I	Overall activity was modest, with one partial response among 13 RECIST-evaluable patients and PSA50 responses in 21%; however, two remissions exceeded 12 months. Toxicity was substantial, including dose-limiting toxicities in 18% and one fatal IEC-HS/HLH event.	Slovin et al. (2026)
NCT02744287	PSCA	Metastatic pancreatic and prostate cancers	Phase I	Objective responses were limited to two patients with mCRPC, one unconfirmed; two dose-limiting toxicities and two treatment-related deaths occurred in the highest-dose cohort, leading to study termination before an RP2D was established.	Stein et al. (2024)
NCT04483778	B7-H3	Relapsed or refractory pediatric and young-adult non-CNS solid tumors	Phase I	No objective response followed the first infusion, whereas one PERCIST partial response occurred after a second infusion; low initial CAR T-cell expansion limited activity, while marked expansion after redosing was accompanied by CRS and dose-limiting transaminitis.	Pinto et al. (2024)
NCT04185038	B7-H3	Diffuse intrinsic pontine glioma (published cohort)	Phase I	Repeated intraventricular dosing was feasible, but objective radiographic responses were rare (1 of 18 evaluable patients) and most patients achieved stable disease; efficacy remained heterogeneous without a validated predictor of response.	Vitanza et al. (2025)
NCT02107963	GD2	Osteosarcoma and neuroblastoma	Phase I	Antitumor responses were limited and CAR T-cell expansion varied markedly; poor expansion was associated with exhausted infusion products and unfavorable myeloid states, implicating both product fitness and host immune context.	Kaczanowska et al. (2024)
NCT04438083	CD70	Advanced clear-cell renal cell carcinoma	Phase I	Disease control was frequent but consisted mainly of stable disease (75%); ORR was only 6% despite one exceptional durable complete response, and circulating CAR T-cells generally declined to undetectable levels by day 28.	Pal et al. (2024)
NCT03392064	DLL3	Relapsed or refractory small-cell lung cancer	Phase I	Among four evaluable patients, one confirmed partial response and two cases of stable disease were observed; the very small cohort precluded reliable assessment of response durability or a dominant biological resistance mechanism.	Zhou et al. (2024)

### 2.3 Interconnected biological barriers explain the failure of durable control

Clinical specimens indicate that loss of control reflects interconnected barriers. In recurrent glioblastoma, EGFRvIII CAR T-cells reached intracranial tumors, but post-treatment samples showed antigen loss together with increased PD-L1, IDO1, and regulatory T-cell signals (O'Rourke et al., 2017). In a separate study of newly diagnosed EGFRvIII-positive glioblastoma, repeated EGFRvIII CAR T-cell infusions combined with pembrolizumab did not demonstrate clinical efficacy (Bagley et al., 2024). These observations shift attention to the mechanisms that prevent early CAR T-cell activity from translating into durable tumor control. Incomplete infiltration, heterogeneous antigen exposure, transient expansion, adaptive immunosuppression, and limited persistence reinforce one another, shaping the stimulation history and fate of infused cells and motivating the failure–pressure–fate framework developed below.

## 3 Clinical failure routes and pressure states shaping CAR T-cell engagement

Solid-tumor CAR T-cell failure is often described as a collection of independent barriers, but these barriers are more informative when considered in terms of how they shape tumor exposure and sustained CAR T-cell function. Three failure routes are particularly relevant: insufficient trafficking and penetration limit spatial coverage; heterogeneous recognition and antigen escape prevent complete tumor clearance; and residual disease sustains CAR engagement within a metabolically, immunologically, and mechanically adverse microenvironment (Fig. 2). The relative importance of these routes, however, differs across tumor types and may change as individual bottlenecks are overcome. In glioblastoma, antigen heterogeneity, antigen loss, and myeloid-rich immunosuppression become particularly important once tumor access is achieved. Pancreatic cancer is characterized by substantial stromal and trafficking constraints, although recent locoregional and tumor–stroma co-targeting strategies suggest that these barriers can be partially bypassed or remodeled, thereby revealing additional limitations in antigen coverage and persistence. Neuroblastoma further illustrates that the dominant bottleneck can vary with CAR target, disease context, and product design. Thus, solid tumors do not impose a uniform pressure landscape; instead, distinct and evolving combinations of failure routes can converge on similar downstream CAR T-cell dysfunction (Table 3).

**Fig. 2:**
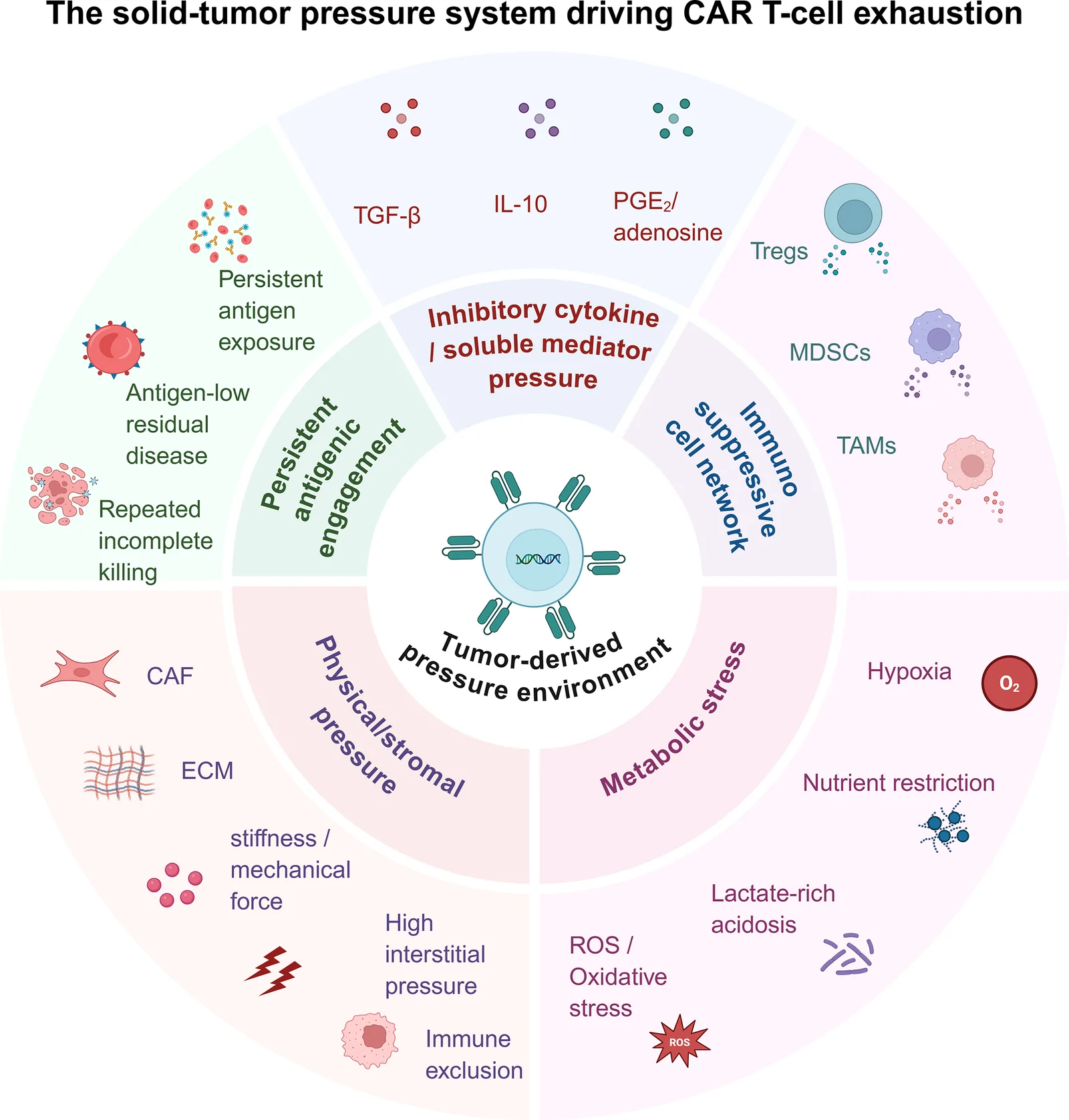
**Interacting tumor pressures that shape CAR T-cell dysfunction and exhaustion-prone fate.** CAR T-cells in solid tumors encounter multiple, interconnected pressure modules that act simultaneously and can reinforce one another. Metabolic stress, including hypoxia, nutrient restriction, lactate-rich acidosis and ROS-associated oxidative stress, reduces metabolic fitness and the capacity to sustain repeated effector responses. Immunosuppressive cellular networks formed by Tregs, MDSCs and TAMs, together with inhibitory soluble mediators such as TGF-β, IL-10, PGE2 and adenosine, further suppress CAR T-cell expansion and function. Physical and stromal barriers generated by CAFs and the extracellular matrix increase tissue stiffness and interstitial pressure and promote immune exclusion, thereby limiting productive tumor access and engagement. Persistent antigenic engagement, arising from continued antigen exposure, antigen-low residual disease and repeated incomplete killing, maintains repetitive CAR stimulation. These pressure modules do not represent isolated mechanisms; rather, they collectively shape the post-infusion tumor environment in which CAR signals are repeatedly interpreted and thereby shape the signaling context in which persistent CAR engagement can promote CAR T-cell dysfunction and progression toward exhaustion-associated fate states.

**Table 3:** Major barriers to CAR T-cell activity in selected solid tumors.

Tumor type	Main barriers	Key features	References
Glioblastoma	Antigen heterogeneity; local immunosuppression	Variable antigen expression and antigen loss can reduce tumor coverage. Local immunosuppression may further limit CAR T-cell activity even when intracranial delivery improves tumor access.	(Choi et al., 2024; O'Rourke et al., 2017)
Pancreatic ductal adenocarcinoma	Stromal exclusion; poor trafficking; immunosuppression	Dense stroma and extracellular matrix restrict CAR T-cell infiltration. Nutrient limitation and fibroblast- or myeloid-mediated suppression may further reduce CAR T-cell function.	(Wehrli et al., 2024; Xiao et al., 2023)
Neuroblastoma	Myeloid suppression; variable trafficking and antigen coverage	MDSCs and related chemokine signals can reduce CAR T-cell persistence in some models. However, durable responses with GD2 CAR T-cells show that these barriers vary with the target and CAR design.	(Del Bufalo et al., 2023; Li et al., 2025)
Gastric/gastroesophageal junction cancer	Antigen heterogeneity; local immunosuppression	Variable CLDN18.2 expression may leave part of the tumor unrecognized. Satri-cel has shown clinical activity, but limited response durability does not by itself identify the main resistance mechanism.	(Qi et al., 2024, 2025; Zhou, 2025)
Prostate cancer	TGF-β-mediated suppression; variable tumor access and antigen expression	TGF-β can suppress CAR T-cell activity, while tumor access and target expression may also limit efficacy. TGF-β-resistant CAR T-cells show biological activity, but blocking one pathway may not be sufficient for durable control.	(Dorff et al., 2024; Narayan et al., 2022)
Ovarian cancer	Antigen heterogeneity; immunosuppression; metabolic stress	Variable antigen expression, suppressive ascites and metabolic stress may reduce CAR T-cell persistence and function. Widespread peritoneal disease can also make complete tumor coverage difficult.	(Cutri-French et al., 2024; Ji et al., 2026)

### 3.1 Insufficient exposure

CAR T-cells must reach, extravasate into and distribute across antigen-bearing tumor regions before target recognition can translate into effective tumor clearance. Chemokine mismatch is one component of this problem. In direct CAR T models, forced expression of CXCR1 or CXCR2 enabled cells to exploit tumor-derived IL-8 gradients, increasing migration and persistence within glioblastoma, ovarian and pancreatic tumor models and improving antitumor activity (Jin et al., 2019). Vascular access provides a second gate: VEGFR2-directed CAR-engineered lymphocytes accumulated in vascularized tumors and inhibited growth across multiple syngeneic tumor models, providing early proof that the tumor vascular compartment can be therapeutically exploited to improve access (Chinnasamy et al., 2010). After extravasation, dense extracellular matrix remains a major obstacle. Caruana and colleagues showed that ex vivo-expanded CAR T-cells had impaired heparanase activity and that restoring heparanase enhanced matrix degradation, tumor infiltration and antitumor efficacy in stroma-rich tumors (Caruana et al., 2015).

These studies define insufficient exposure as a problem of spatial coverage. Chemokine mismatch, dysfunctional vasculature and stromal exclusion leave antigen-bearing regions incompletely surveyed. Their immediate consequence is not exhaustion itself, but incomplete debulking and an uneven geography of subsequent antigen engagement.

### 3.2 Incomplete recognition and clearance

Physical access is insufficient if CAR T-cells cannot recognize and eliminate the full malignant population. Solid-tumor antigens vary across cells and lesions, and many are also expressed at lower levels in normal tissues. Clinical evidence from EGFRvIII-directed CAR T-cell therapy in recurrent glioblastoma illustrates the dynamic nature of this problem: CAR T-cells trafficked to intracranial tumors, yet post-treatment specimens showed loss or reduction of EGFRvIII together with adaptive changes in the local immune environment (O'Rourke et al., 2017). Antigen loss can also be shaped by the therapy itself. Hamieh and colleagues demonstrated that CAR-mediated trogocytosis transfers target antigen from tumor cells to CAR T-cells, reducing target density while promoting fratricide and exhaustion-like dysfunction (Hamieh et al., 2019). Although established in a hematologic model, this provides direct CAR T evidence that antigen availability can be dynamically altered during immune attack.

Recognition of residual antigen is also governed by CAR design. Majzner et al. showed that the antigen-density threshold required for CAR activation can be shifted by costimulatory architecture, CD3ζ ITAM configuration and other receptor features (Majzner et al., 2020). Affinity imposes a related trade-off. In a recent mesothelin CAR study, systematic affinity tuning showed that high-affinity constructs expanded extensively in normal tissues, whereas a lower-affinity variant preferentially localized to MSLN-high tumors, improved tumor control and reduced on-target, off-tumor toxicity (Yang et al., 2024). Together, these studies place antigen heterogeneity, antigen density and receptor sensitivity within the same clearance problem: insufficient sensitivity leaves antigen-low tumor cells behind, whereas excessive sensitivity narrows the therapeutic window and broadens the range of low-density encounters capable of sustaining CAR signaling.

### 3.3 Persistent engagement under hostile conditions

Once residual disease persists, repeated antigen encounter creates a sustained stimulation context, but the consequences of this stimulation depend on determinants operating at different stages. Before infusion, manufacturing establishes the developmental, metabolic and functional state of the CAR T-cell product, while receptor architecture further shapes its intrinsic signaling susceptibility. After infusion, these pre-existing properties are challenged by tumor-derived pressures, including metabolic restriction, immunosuppressive signals and stromal or mechanical constraints. These layers are considered sequentially below to distinguish pre-infusion susceptibility from the post-infusion pressure environment.

#### 3.3.1 Pre-infusion susceptibility: manufacturing and design set the vulnerability threshold

Manufacturing does not constitute a tumor-derived pressure. Instead, it establishes the cellular starting state from which subsequent antigenic and microenvironmental pressures are interpreted. Conventional activation and prolonged ex vivo expansion can progressively consume differentiation and proliferative reserve before infusion, whereas rapid or minimally expanded manufacturing preserves a larger fraction of naïve- and stem-like cells and improves subsequent expansion and persistence (Dickinson et al., 2023; Ghassemi et al., 2022). Longitudinal profiling of CAR T-cell production further shows that this remodeling is dynamic: shorter culture retains more stem-like and metabolically active populations, whereas prolonged expansion enriches more differentiated and exhaustion-associated states (Cadinanos-Garai et al., 2025). Thus, manufacturing duration affects not only the time required to deliver the product, but also the differentiation state in which CAR T-cells first encounter tumor-derived pressure.

Starting-cell composition provides a second layer of fate preconditioning. CAR T products generated from preselected naïve/stem-memory T cells display greater proliferative reserve, improved persistence and a more favorable memory/exhaustion phenotype than products derived from unselected bulk T cells, even when their immediate effector signature is less pronounced (Arcangeli et al., 2022). Clinical and lineage-tracing studies further indicate that this pre-infusion composition is not erased after transfer. Stem-memory clones present at low frequency in the infusion product can contribute disproportionately to both early expansion and long-term CAR T-cell persistence, while premanufacture TCF7-associated stemness programs are linked to more durable in vivo persistence (Biasco et al., 2021; Chen et al., 2021). Manufacturing therefore shapes cell yield, short-term cytotoxic potency, and the clonal and developmental substrate available for subsequent in vivo expansion.

Manufacturing conditions can also actively reshape this substrate. Inosine exposure or adenosine-deaminase engineering during production induces a stemness-associated metabolic and epigenetic program, characterized by reduced glycolytic dependence, increased mitochondrial and glutamine-supported metabolic capacity, and epigenomic remodeling toward a more stem-like state (Klysz et al., 2024). This example is mechanistically distinct from rapid manufacturing or starting-cell selection. Rapid manufacturing primarily limits the loss of an early differentiation state, and starting-cell selection enriches the regenerative compartment present at the outset, whereas metabolic conditioning can actively reprogram the product toward a more favorable pre-infusion state. Receptor design adds a further layer: CARs prone to excessive tonic signaling can impose chronic activation during ex vivo culture and thereby lower the threshold for subsequent dysfunction (Chen et al., 2023; Long et al., 2015).

These manufacturing-imprinted states are neither uniformly transient nor necessarily permanent. Functional activation and metabolic conditioning may remain adjustable after infusion, whereas advanced differentiation, depletion of stem-like precursors and progressively stabilized epigenetic programs are more likely to persist by limiting the repertoire of states available for subsequent expansion. Clinical studies support this distinction. CAR T populations that dominate early expansion and those that sustain later persistence can arise from phenotypically distinct precursors already present in the infusion product, indicating that post-infusion tumor exposure acts on a heterogeneous pre-existing cellular substrate rather than completely resetting CAR T-cell identity (Cao et al., 2025). Accordingly, manufacturing imprint is best viewed as a continuum ranging from reversible functional conditioning to increasingly stable developmental and epigenetic bias.

Within the pressure–fate model, manufacturing and receptor design therefore define a vulnerability threshold rather than a separate exhaustion pressure. Products that retain TCF7-associated stemness, proliferative reserve, metabolic fitness and epigenetic plasticity are better positioned to tolerate repeated antigen encounter, whereas products shaped by prolonged activation, advanced differentiation, metabolic limitation or tonic signaling may enter dysfunction earlier. Direct evidence that individual manufacturing strategies themselves restore NFAT–AP-1 balance remains limited; the more defensible interpretation is that manufacturing determines the cellular context in which later CAR signaling is decoded. Once persistent antigenic and microenvironmental pressures are encountered after infusion, a vulnerable starting state may more readily progress toward NFAT-biased signaling, TOX/NR4A reinforcement, metabolic dysfunction and progressive epigenetic restriction. Manufacturing therefore sets the susceptibility with which subsequent tumor-derived pressure is converted into exhausted fate (Fig. 1).

#### 3.3.2 Persistent and dysregulated CAR signaling

CAR signaling is shaped by receptor architecture even before antigen encounter. Antigen-independent tonic signaling is not uniformly detrimental but follows a non-monotonic, optimal-window relationship with CAR T-cell fitness. Long et al. showed that excessive tonic signaling caused by spontaneous scFv clustering promotes premature exhaustion, whereas Chen et al. demonstrated that reducing tonic signaling in high-tonic CARs alleviates dysfunction and that increasing tonic signaling in a weakly signaling CD19 CAR improves persistence and antitumor activity. These findings support a “PEAK” model in which intermediate signaling is optimal (Chen et al., 2023; Long et al., 2015). This concept has also become experimentally tunable: CAR-Toner uses AI-assisted analysis of scFv positively charged patches to predict and optimize tonic-signaling propensity (Qiu et al., 2024), while a fulvestrant-inducible phase-separation CAR enables reversible pharmacological enhancement of CAR clustering and signaling, improving antigen sensitivity and antitumor function (Huang et al., 2026). Thus, tonic signaling should be maintained within an optimal range rather than simply suppressed.

#### 3.3.3 Metabolic and suppressive co-pressure

Persistent CAR signaling becomes increasingly difficult to sustain when receptor engagement continues under metabolic and immunological restriction. Direct CAR T studies support treating these factors as co-pressures rather than isolated exhaustion programs. Hypoxia-responsive CAR T-cells engineered to confine CAR expression to hypoxic conditions displayed reduced exhaustion-associated features and improved activity in solid-tumor models, directly linking oxygen context to CAR T functional state (Zhu et al., 2024). Nutrient access is similarly important. GLUT1 overexpression reprogrammed CAR T-cell metabolism, improving glucose utilization, respiratory fitness and antitumor potency while reducing exhaustion-associated features (Guerrero et al., 2024). Together, hypoxia, nutrient restriction, lactate-rich acidosis and oxidative stress constrain the bioenergetic reserve available for repeated killing (Liu et al., 2026).

Soluble and cellular suppressive pathways further lower this reserve. Newick et al. disrupted PKA anchoring and thereby increased CAR T-cell resistance to adenosine- and PGE2-mediated suppression, improving trafficking and antitumor efficacy (Newick et al., 2016). The functional relevance of TGF-β pressure is supported by clinical testing of TGF-β-insensitive PSMA CAR T-cells (Narayan et al., 2022). Myeloid populations provide complementary pressure. MDSCs impaired anti-CEA CAR T efficacy in a liver-metastasis model (Burga et al., 2015), whereas CD163-positive macrophages suppressed CAR T activity through an IFN-γ-induced PD-L1 feedback loop that could be countered by PD-L1 blockade (Yamaguchi et al., 2022).

These pressures need not independently establish an exhaustion program. Instead, CAR signaling persists while nutrient availability, mitochondrial reserve, redox control and permissive immune signaling deteriorate, making repeated engagement progressively less able to sustain proliferation, cytokine production and serial killing.

#### 3.3.4 Stromal and mechanical pressure

The stromal compartment can continue to influence CAR T-cells after infiltration. This role is distinct from the spatial-exclusion function described above. A recent study identified Osr2 as a biomechanically responsive checkpoint induced by combined antigen-receptor and mechanical-force signaling through a Piezo1–calcium–CREB pathway; Osr2 recruited HDAC3 and suppressed cytotoxic programs, and Osr2 deletion also improved CAR T-cell persistence and antitumor activity in solid-tumor models (Zhang et al., 2024). This provides direct CAR T validation for a mechanical contribution to dysfunction, although the evidence base is smaller than that for chronic signaling or metabolic suppression. Mechanical pressure is therefore best positioned as an emerging amplifier after entry, rather than as a fully established independent exhaustion axis.

## 4 Mechanistic conversion of pressure into exhausted fate

The upstream pressures described above do not map one-to-one onto a single exhaustion pathway. Their common consequence is that CAR T-cells continue to receive antigen-receptor signals while the cellular context required to translate those signals into productive activation progressively deteriorates. Exhaustion can therefore be viewed as a conversion process: persistent or dysregulated signaling first changes how CAR signals are interpreted; this altered output is reinforced by exhaustion-associated transcriptional programs and declining metabolic fitness; and repeated stimulation eventually remodels chromatin and DNA methylation in ways that restrict developmental plasticity. The endpoint is not a uniform exhausted population, but a spectrum of CAR T-cell states that differ in proliferative reserve, reversibility and therapeutic responsiveness.

### 4.1 Signal-logic imbalance: persistent signaling uncouples NFAT from productive AP-1 cooperation

Productive T-cell activation requires coordinated transcriptional signaling rather than NFAT activity alone. Calcium–calcineurin signaling promotes NFAT nuclear translocation, whereas MAPK-dependent pathways activate AP-1, a dimeric transcription-factor complex formed mainly by JUN- and FOS-family proteins. During acute activation, NFAT and AP-1 cooperate at composite regulatory elements to support proliferation, cytokine production and cytotoxic effector programs.

Mechanistic evidence that disruption of this cooperation can redirect T-cell signaling initially came from conventional CD8^+^ T cells. Martinez et al. engineered an NFAT1 variant that retained transcriptional activity but could no longer cooperate normally with AP-1. In primary CD8^+^ T cells, this AP-1-independent NFAT activity induced a transcriptional program overlapping with that of exhausted CD8^+^ T cells and impaired productive effector responses (Martinez et al., 2015). These findings established an important principle: sustained NFAT activity does not inherently determine productive activation; its transcriptional output depends on the partner context in which NFAT operates.

The functional relevance of this principle was subsequently demonstrated directly in CAR T-cells. c-Jun is a canonical JUN-family component of AP-1. Lynn et al. found that exhausted human CAR T-cells displayed altered AP-1-associated regulation and functional c-Jun insufficiency. Engineering these cells to overexpress c-Jun enhanced proliferation and cytokine production, reduced terminal differentiation and improved antitumor activity under exhaustion-inducing conditions (Lynn et al., 2019). These findings do not support a simple model in which AP-1 is globally lost during exhaustion. Rather, chronic CAR stimulation appears to alter the composition and functional output of the AP-1 network, reducing the productive cooperation between NFAT and AP-1 that normally supports sustained effector function.

Thus, the earliest intracellular transition toward exhaustion is best described as NFAT-biased signaling with insufficient productive AP-1 cooperation. The shorthand “NFAT-high/AP-1-low” does not adequately capture this network-level change. Persistent CAR engagement changes how receptor signals are transcriptionally interpreted, creating an exhaustion-prone output that can subsequently be reinforced by downstream transcriptional programs (Fig. 3).

**Fig. 3:**
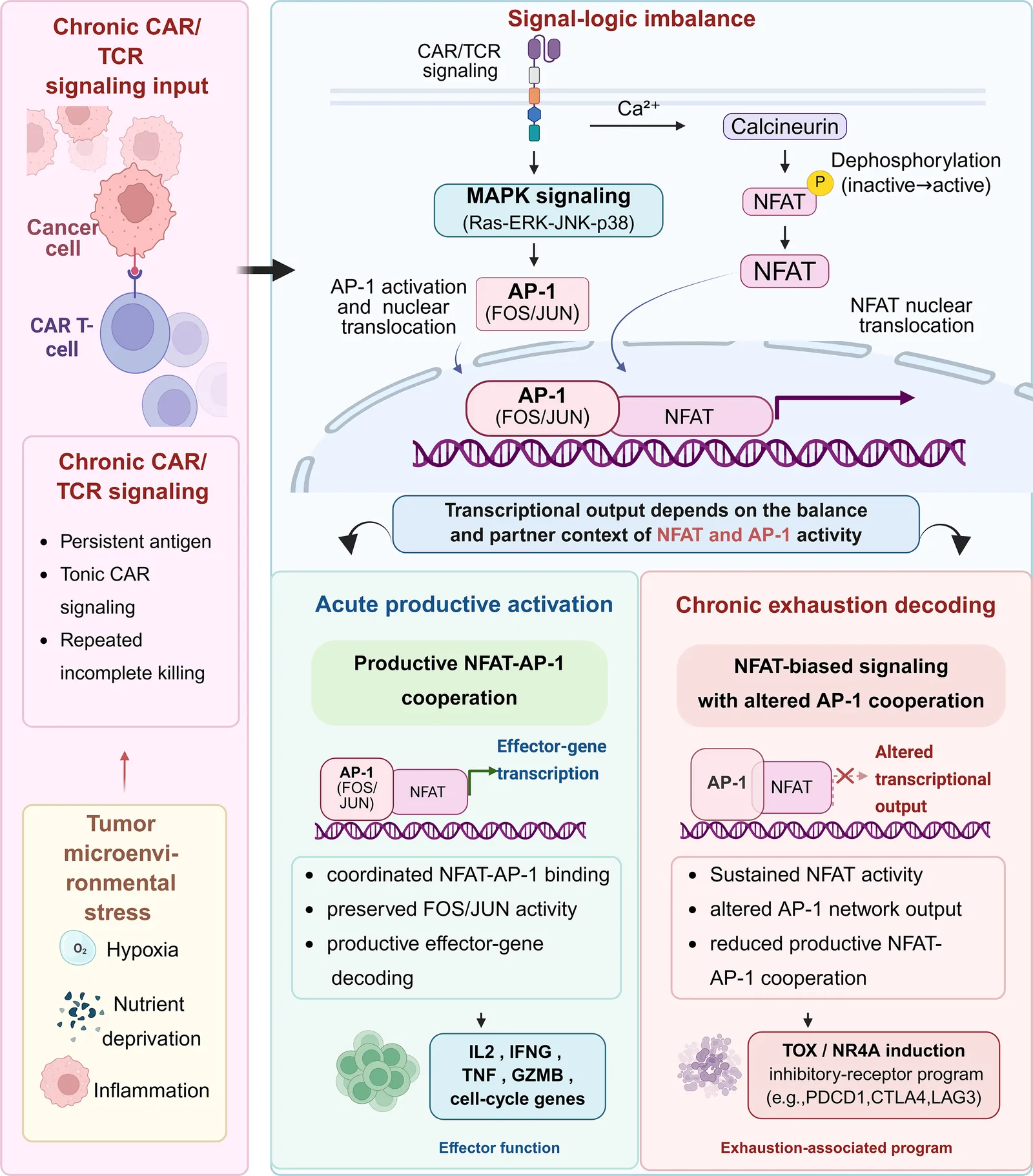
**Chronic CAR signaling drives NFAT–AP-1 signal-logic imbalance.** Persistent antigen exposure, tonic CAR signaling and repeated incomplete tumor killing sustain chronic CAR/TCR stimulation in solid tumors, while microenvironmental stresses such as hypoxia, nutrient deprivation and inflammation further reinforce this signaling context. CAR/TCR engagement activates both calcium–calcineurin–NFAT signaling and MAPK-dependent AP-1 signaling. Calcineurin-mediated NFAT dephosphorylation promotes NFAT nuclear translocation, whereas MAPK signaling activates AP-1 complexes composed mainly of FOS- and JUN-family proteins. During acute productive activation, coordinated NFAT–AP-1 binding supports effector-gene transcription, including cytokine, cytotoxic and proliferative programs. Chronic stimulation alters AP-1 composition and functional output, reducing productive cooperation with NFAT. The resulting NFAT-biased transcriptional decoding promotes TOX/NR4A induction and inhibitory-receptor-associated programs, establishing an exhaustion-prone state that is subsequently reinforced by downstream transcriptional, metabolic and epigenetic mechanisms.

### 4.2 Transcriptional and metabolic reinforcement: NR4A and TOX networks consolidate dysfunctional output

Once CAR signaling is repeatedly decoded in an exhaustion-prone context, dysfunction is reinforced by transcriptional networks that are themselves induced by chronic activation. NR4A family members provide some of the strongest direct evidence for this step in solid-tumor CAR T-cells. Chen et al. showed that exhausted tumor-infiltrating CAR T-cells displayed increased NR4A expression and NR4A-associated chromatin accessibility downstream of NFAT. Deleting all three NR4A family members shifted CAR T-cells toward an effector-associated transcriptional and chromatin state, improved tumor regression and prolonged survival in solid-tumor models (Chen et al., 2019). This genetic rescue provides direct evidence that NR4A contributes functionally to the dysfunctional program.

TOX and TOX2 provide a complementary layer of reinforcement. In tumor-infiltrating CAR T-cells, Seo et al. showed that TOX and TOX2 cooperate with NR4A-associated programs to impose exhaustion, whereas perturbation of these factors increased cytokine expression and reduced inhibitory-receptor features (Seo et al., 2019). These findings shift the model away from a simple linear pathway in which NFAT activates a single “exhaustion gene”. Instead, persistent signaling recruits a broader regulatory network in which NR4A, TOX/TOX2 and differentiation-associated transcription factors collectively reshape the balance between effector and dysfunctional programs. Consistent with this network view, combined disruption of PRDM1 and NR4A3 promoted TCF7-associated CAR T-cell stemness and suppressed exhaustion-associated states more effectively than either perturbation alone (Jung et al., 2022).

Network compensation within this regulatory layer is further illustrated by Yin et al. In glioma CAR T-cells, NR4A3 suppression initially enhanced activity, but the benefit weakened during prolonged tumor exposure as FOS expression declined. Restoring FOS together with NR4A3 suppression improved effector function and tumor clearance (Yin et al., 2025). This finding places NR4A3–FOS crosstalk within the downstream transcriptional reinforcement network rather than the initial NFAT–AP-1 signal-decoding step.

Transcriptional reinforcement also becomes coupled to metabolic fitness. This connection should be distinguished from the hypoxia, nutrient restriction and acidosis discussed in the preceding section: those are external metabolic pressures, whereas the issue here is whether the intracellular exhaustion program itself leaves CAR T-cells progressively less capable of meeting energetic demand. In repeatedly stimulated human HER2 CAR T-cells, Nakagawara et al. showed that NR4A ablation enhanced mitochondrial oxidative phosphorylation, preserved less differentiated features, increased intratumoral persistence and improved antitumor activity in solid-tumor models (Nakagawara et al., 2024). This provides direct evidence that transcriptional state and mitochondrial competence are functionally coupled in chronically stimulated CAR T-cells.

The resulting process is therefore self-reinforcing. Persistent signaling reinforces NR4A- and TOX-associated dysfunctional programs, while NR4A-dependent regulation also becomes coupled to declining mitochondrial competence. The increasingly constrained cell is then less able to convert subsequent antigen encounters into proliferation, cytokine production and serial killing. This should not be interpreted as a rigid sequence in which transcriptional changes always precede metabolic decline. Rather, transcriptional and metabolic dysfunction reinforce one another as chronic stimulation continues.

With time, however, this state is no longer maintained by transcription factors and metabolism alone. Repeated activation begins to alter the regulatory landscape on which subsequent signals act, shifting exhaustion from a predominantly functional and transcriptional state toward a progressively fate-restricted one.

### 4.3 Epigenetic restriction: repeated dysfunction narrows the range of recoverable states

The transition from transcriptional dysfunction to epigenetic restriction provides a mechanistic explanation for why early CAR T-cell dysfunction may remain recoverable whereas late exhaustion becomes increasingly difficult to reverse. Human CAR T-cells undergoing exhaustion acquire extensive changes in chromatin accessibility and enhancer–gene relationships. Gennert et al. profiled exhaustion-prone human CAR T-cells across their development and identified dynamic remodeling of accessible chromatin and enhancer regulation, demonstrating that exhaustion alters not only which genes are expressed at a given moment but also the regulatory architecture available for future responses (Gennert et al., 2021). Importantly, epigenetic restriction may begin well before terminal dysfunction becomes apparent. Longitudinal analysis of clinical CAR T-cells identified early DNA-methylation remodeling and repression of memory-associated loci such as TCF7 and LEF1 after infusion, while prolonged ex vivo expansion has also been associated with progressive methylation changes linked to reduced long-term functional potential (Salz et al., 2023; Zebley et al., 2021). These observations suggest that the window for preserving epigenetic plasticity may already begin during manufacturing and continue to narrow after repeated antigen exposure.

DNA methylation provides stronger causal evidence that this regulatory remodeling can alter CAR T-cell fate. Prinzing et al. showed that deleting DNMT3A preserved a stem-like epigenetic program during prolonged antigen stimulation, maintained proliferative and functional capacity and enhanced antitumor activity (Prinzing et al., 2021). This finding is important because it separates epigenetic restriction from a passive consequence of exhaustion: manipulating the methylation machinery changed the trajectory of dysfunction itself. These findings implicate DNMT3A-dependent methylation in the progressive restriction of developmental programs associated with stemness, memory, and sustained proliferation during repeated CAR stimulation. Importantly, permanent gene disruption is not the only way to influence this trajectory. Short, low-dose decitabine exposure has been reported to promote a more memory-like CAR T-cell state, while targeting the H3K9 methyltransferase SUV39H1 similarly enhanced memory-associated chromatin accessibility, persistence and responses to repeated tumor challenge in experimental CAR T-cell systems (Jain et al., 2024; López-Cobo et al., 2024; Wang et al., 2021). These studies broaden the concept of epigenetic intervention from fixed gene deletion toward pharmacological or chromatin-level modulation.

Epigenetic restriction is nevertheless not synonymous with absolute irreversibility. Weber et al. used regulatable CAR systems to interrupt signaling after exhaustion had developed and showed that transient rest restored substantial functional capacity together with broad transcriptional and epigenetic remodeling (Weber et al., 2021). This experiment is particularly informative because the intervention removed persistent CAR signaling rather than simply adding another stimulatory signal. It therefore provides causal evidence for a window in which an exhaustion-associated regulatory state can still be redirected. However, functional recovery should not be equated with complete epigenetic resetting. Studies of chronically stimulated antigen-specific CD8 T cells have shown that removal of persistent antigen can restore aspects of phenotype and function while leaving exhaustion-associated chromatin scars and impaired recall responses (Abdel-Hakeem et al., 2021; Hensel et al., 2021; Yates et al., 2021). Although these studies were not performed in CAR T-cells, they provide an important mechanistic boundary for interpreting reversibility.

This reversibility is likely to be stage dependent. TET2 further illustrates that epigenetic regulators do not operate as simple beneficial or detrimental switches. Dimitri et al. showed that TET2 regulates early and late transitions during exhausted CD8 T-cell differentiation and also limits CAR T-cell function, linking active DNA-demethylation machinery to the progression between exhaustion-associated states (Dimitri et al., 2024). Together, the Gennert, Prinzing, Weber and Dimitri studies establish a useful hierarchy of evidence: CAR T exhaustion is accompanied by regulatory-landscape remodeling; manipulating an epigenetic regulator can alter exhaustion trajectory; interruption of chronic signaling can reopen part of that landscape; and the effect of epigenetic regulators depends on the stage of differentiation. The therapeutic window is therefore better defined by residual epigenetic plasticity than by elapsed treatment time alone: intervention is most likely to achieve durable redirection while progenitor- or memory-associated regulatory programs remain accessible.

The central consequence is a progressive loss of epigenetic plasticity. Early dysfunctional cells may still retain regulatory access to memory- and progenitor-associated programs and can therefore be redirected when chronic pressure is relieved. With sustained stimulation, however, repeated transcriptional activity and epigenetic remodeling narrow the available developmental routes. At this point, checkpoint blockade or cytokine support may increase short-term activity without fully restoring a durable memory-like state. The relevant biological boundary is the preservation of regulatory architecture that supports self-renewal and renewed differentiation; inhibitory-receptor expression alone is insufficient to define this state.

This therapeutic opportunity must nevertheless be balanced against safety. Epigenetic regulators control not only exhaustion-associated programs but also proliferation, clonal fitness and long-term cellular identity. In a specific CAR and BATF3 context, biallelic TET2 disruption permitted antigen-independent CAR T-cell clonal expansion, systemic tissue infiltration and acquisition of secondary somatic alterations (Jain et al., 2023). These consequences are not inevitable, because long-term persistence of Dnmt3a-, Tet2- or Asxl1-deficient T cells without malignant transformation has also been observed experimentally, indicating that the risk is context dependent (Kang et al., 2024). Accordingly, deeper and permanent epigenetic manipulation should incorporate greater controllability and surveillance. Dimitri et al. incorporated a functional safety switch into TET2-targeted CAR T-cells, providing a mechanism for selective elimination of the edited population if required (Dimitri et al., 2024). Emerging targeted epigenome-editing approaches may further reduce the need for broad, irreversible disruption of chromatin regulators, although their long-term safety remains to be established. Thus, the translational goal should be controlled preservation or reopening of useful regulatory states rather than unrestricted erasure of exhaustion-associated epigenetic programs.

### 4.4 Fate continuum: from plastic stem-like states to fate-restricted dysfunction

The cumulative effect of altered signal decoding, transcriptional–metabolic reinforcement and epigenetic restriction is best represented as a continuum of CAR T-cell states rather than a single exhausted endpoint. At one end of this spectrum are Tpex-like CAR T-cells that retain proliferative reserve and the capacity to generate more differentiated progeny. At the other end are terminal Tex-like CAR T-cells with limited proliferative and regenerative potential. Between these extremes are intermediate or transitory states in which effector capacity has declined but developmental plasticity has not yet been fully lost. The differentiation trajectories of these populations may differ from those established in endogenous antigen-specific T cells, reflecting the influence of CAR architecture, costimulatory signaling and manufacturing history (Fig. 4).

**Fig. 4:**
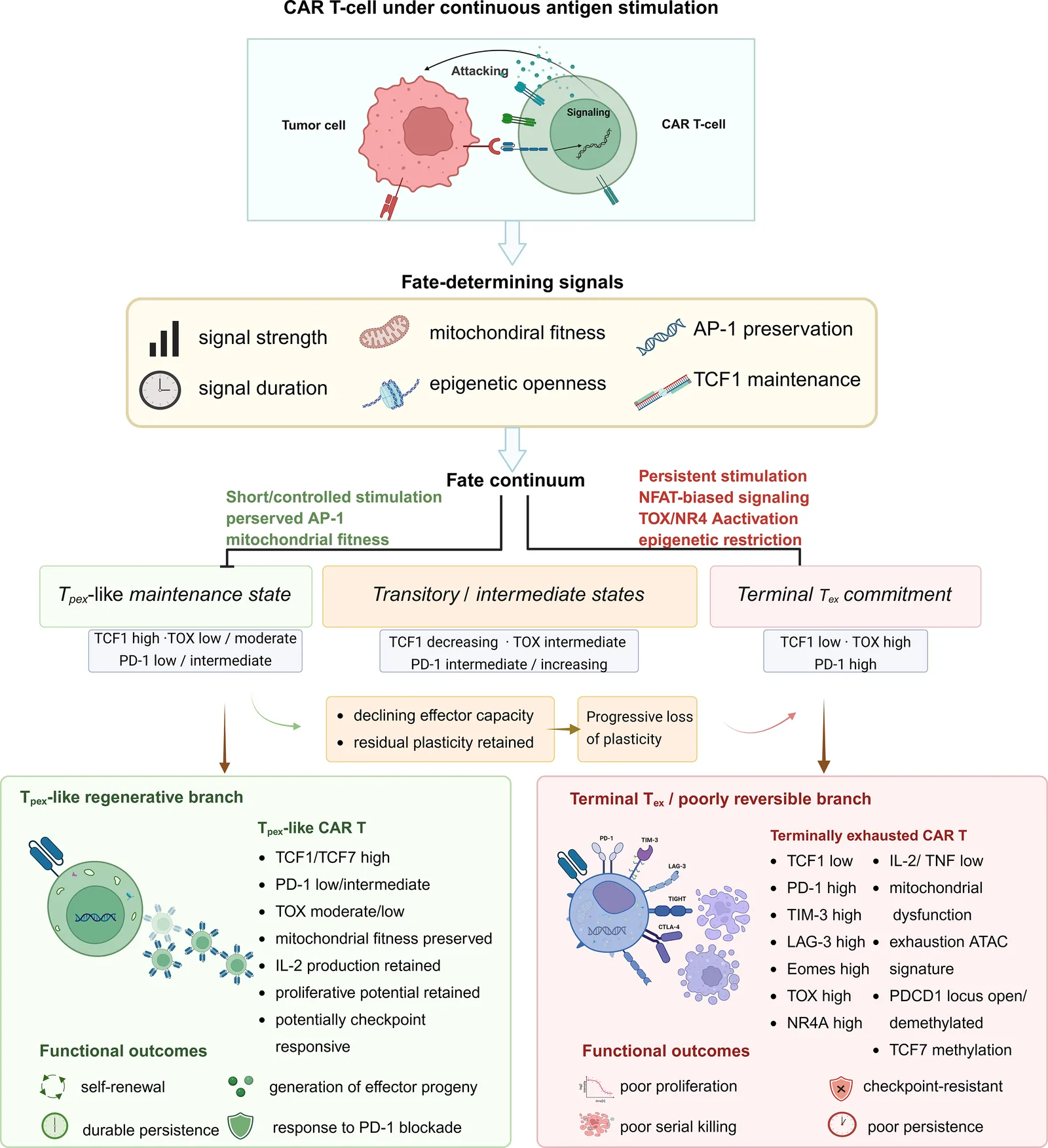
**CAR T-cell fate continuum from Tpex-like regenerative states to fate-restricted dysfunction.** Continuously stimulated CAR T-cells progress along a fate continuum shaped by signal strength and duration, mitochondrial fitness, AP-1 activity, epigenetic accessibility, and maintenance of TCF1-associated progenitor programs. Under short or controlled stimulation, preserved AP-1 activity, mitochondrial fitness and TCF1 expression favor maintenance of a Tpex-like, relatively plastic state. These cells are characterized by high TCF1/TCF7, low-to-intermediate PD-1 and low-to-moderate TOX, retain proliferative capacity and IL-2 production, preserve mitochondrial fitness and may retain responsiveness to checkpoint blockade in appropriate contexts. Functionally, the Tpex-like branch supports self-renewal, generation of effector progeny and durable persistence. With persistent stimulation, however, NFAT-biased signaling, TOX/NR4A activation, mitochondrial dysfunction and progressive epigenetic restriction drive cells through intermediate states in which effector capacity declines and residual plasticity is progressively lost. Terminally exhausted CAR T-cells are characterized by low TCF1 together with high PD-1, TIM-3, LAG-3, TOX, NR4A and Eomes, reduced IL-2/TNF production, mitochondrial dysfunction and exhaustion-associated epigenetic features, including TCF7 methylation and an accessible or demethylated PDCD1 locus. These fate-restricted cells display poor proliferation, impaired serial killing, reduced persistence and limited checkpoint responsiveness. Representative marker trends are shown to aid interpretation of the continuum and should be understood as qualitative state-associated patterns rather than universal quantitative thresholds.

Recent CAR T studies provide strong direct evidence for a plastic, regeneration-competent state. Two independent studies published in Nature in 2024 identified FOXO1 as a major regulator of durable CAR T-cell identity. Doan et al. showed that endogenous FOXO1 promotes memory-associated gene expression and restrains exhaustion in human CAR T-cells; FOXO1 overexpression preserved memory potential, metabolic fitness and function during chronic stimulation and improved persistence and tumor control across multiple models (Doan et al., 2024). Independently, Chan et al. demonstrated that FOXO1 enhances CAR T-cell stemness, metabolic fitness, persistence and antitumor efficacy, including in solid-tumor models (Chan et al., 2024). Together, these studies link durable CAR T-cell competence to preservation of regenerative transcriptional and metabolic programs.

More direct evidence for a progenitor-like intratumoral CAR T-cell compartment has emerged. Snyder et al. showed that AP-1 modulation promoted the establishment of an intratumoral PD-1^+^TCF1^+^ stem-like CAR T-cell reservoir and restored responsiveness to PD-1 blockade, linking precursor-state maintenance to therapeutic responsiveness (Snyder et al., 2026).

CAR T fate is also experimentally tunable along a differentiation hierarchy. Recent work demonstrated that BACH2-dependent programming regulates CAR T-cell lineage state and that manipulating BACH2 alters TCF1-associated stemness, differentiation and antitumor function (Chang et al., 2026). These findings support the existence of a developmental axis along which regenerative potential can be preserved or progressively lost.

At the same time, this fate landscape should not be forced into a strictly linear model. Selli et al. demonstrated that chronic stimulation of CARs carrying different costimulatory domains can generate distinct dysfunctional states; notably, dysfunction driven by 4-1BB-containing CARs differed from canonical exhaustion (Selli et al., 2023). Together, these findings support a heterogeneous CAR T-cell fate landscape in which progenitor-like states may be preserved or progressively lost, while the differentiation trajectories remain dependent on receptor design and stimulation context.

Surface inhibitory receptors alone cannot establish whether a dysfunctional CAR T-cell remains recoverable. A more informative description of fate must consider whether progenitor/memory programs, mitochondrial reserve and epigenetic plasticity are still preserved. These differences in residual plasticity provide the biological rationale for assessing CAR T-cell state alongside the pressure environment. The next section therefore asks which features of pressure and fate can be measured with clinically accessible, translational or investigational approaches.

## 5 A translational assessment framework for pressure and CAR T-cell fate

The distinction between upstream pressure and downstream CAR T-cell fate has translational value, but it should not yet be framed as an established diagnostic system. No validated biomarker panel currently separates reversible dysfunction from fate-restricted exhaustion across solid tumors, and available assays differ substantially in accessibility and biological resolution. A practical framework should therefore distinguish what can be monitored clinically, what can be assessed in translational studies, and what remains investigational. The framework distinguishes failure associated with inadequate CAR T-cell availability, incomplete tumor exposure or recognition, persistent local pressure, and progressive loss of cellular plasticity. It is not intended to define fixed exhaustion categories.

### 5.1 Clinically accessible assessment: product fitness and in vivo kinetics

The most accessible information comes from the infused product and serial peripheral samples. Product assessment can define CAR expression, cellular composition and differentiation state before infusion, providing a baseline estimate of starting fitness. In a phase 1 study of IL-13Rα2 CAR T-cells for recurrent high-grade glioma, products derived from naïve/stem-memory/central-memory populations displayed a less differentiated phenotype and the highest CAR T-cell persistence in peripheral blood compared with the earlier central-memory-derived platform (Brown et al., 2024). These measurements can therefore characterize product quality, but should not be interpreted as a diagnostic definition of exhaustion.

After infusion, longitudinal measurements of CAR T-cell abundance provide a direct readout of expansion and persistence. In patients with H3K27M-positive diffuse midline glioma, Monje et al. monitored GD2 CAR T-cells by CAR-specific quantitative PCR and flow cytometry in blood and cerebrospinal fluid; peripheral CAR T-cells expanded after intravenous infusion and then declined over time (Monje et al., 2025). Such measurements can distinguish failure associated with poor expansion or loss of the cellular product from failure despite continued persistence. Conventional flow cytometry can add information on differentiation, proliferation and inhibitory-receptor expression, but markers such as PD-1 (Shi et al., 2025), TIM-3 or TCF1 should be interpreted in context rather than as stand-alone fate classifiers. Their value is greatest when serial measurements are interpreted against the pre-infusion product and the timing of clinical response or relapse.

### 5.2 Translationally feasible assessment: tumor exposure, antigen geography and local pressure

Peripheral monitoring cannot establish whether CAR T-cells have reached relevant tumor compartments or whether the target antigen remains available. Tumor tissue therefore provides a more informative, although less accessible, layer. In recurrent glioblastoma treated with EGFRvIII-directed CAR T-cells, post-treatment specimens demonstrated CAR T-cell trafficking together with reduced EGFRvIII expression and adaptive changes in the local immune environment (O'Rourke et al., 2017). Paired tissue analysis can therefore reveal recognition and local resistance mechanisms that are invisible from peripheral CAR T-cell counts alone.

Tumor immune contexture can also be evaluated with assays feasible in prospective translational trials. Brown et al. used conventional and three- or four-plex immunohistochemistry in pretreatment glioma specimens and found that higher intratumoral CD3 infiltration was associated with longer overall survival after IL-13Rα2 CAR T-cell therapy (Brown et al., 2024). These approaches can characterize antigen distribution, immune-cell localization and major features of the surrounding microenvironment. Their current role, however, is mechanistic stratification rather than treatment assignment, because spatial heterogeneity, sampling bias and limited access to serial biopsies preclude validated decision thresholds.

### 5.3 Investigational profiling: resolving fate and residual plasticity

The most difficult question is whether dysfunctional CAR T-cells retain sufficient plasticity to recover. Single-cell transcriptomic, multi-omic and epigenetic approaches provide substantially greater resolution than routine phenotyping, but remain investigational as clinical decision tools. In patients with recurrent glioblastoma treated with bivalent EGFR–IL-13Rα2 CAR T-cells, Freeburg et al. combined longitudinal single-cell RNA sequencing of cerebrospinal fluid with profiling of infusion products and matched tumor samples. CAR T-cells acquired activation and exhaustion-associated features after infusion, while distinct remodeling of endogenous NK, regulatory T-cell and myeloid compartments accompanied different clinical outcomes (Freeburg et al., 2026). This demonstrates the capacity of single-cell profiling to resolve response and resistance states in human solid-tumor CAR T-cell therapy without establishing a prospective treatment-selection signature.

Epigenetic profiling extends this analysis to regulatory plasticity. Gennert et al. demonstrated dynamic remodeling of chromatin accessibility and enhancer–gene relationships in exhaustion-prone human CAR T-cells, providing a mechanistic basis for ATAC-based or related fate-state signatures (Gennert et al., 2021). At present, however, such signatures remain candidate biomarkers: CAR design, manufacturing, tumor type, sampling time and analytical pipeline can all alter the measured state, and clinically validated thresholds are lacking.

Taken together, these three levels define a hierarchy of inference. Product and peripheral measurements assess starting fitness, expansion and persistence; tumor-based measurements identify exposure, antigen and local-pressure failure; and single-cell or epigenetic profiling investigates residual plasticity and fate. Their integration provides a translational assessment framework rather than a mature diagnostic system.

## 6 Translating pressure and fate into intervention

The preceding framework separates two questions that are often conflated in solid-tumor CAR T-cell therapy: what is currently limiting tumor control, and whether the existing CAR T-cell population still has sufficient plasticity to benefit from correction of that limitation. This distinction changes how intervention strategies should be organized. Intervention priorities should be determined by the dominant level of failure and the recoverability of the existing CAR T-cell population. Because biomarkers of pressure dominance and fate restriction are not yet prospectively validated, the framework below should be viewed as a rationale for translational prioritization rather than a clinical treatment algorithm.

### 6.1 Relieve the dominant upstream pressure

When CAR T-cells retain functional potential but fail because they cannot adequately reach, recognize or operate within the tumor, the first intervention should address the corresponding upstream bottleneck. Additional stimulation is unlikely to compensate for insufficient tumor exposure. Locoregional administration provides the most clinically advanced example. In recurrent high-grade glioma, repeated intracranial delivery of IL-13Rα2 CAR T-cells was feasible and produced biological and clinical activity in a subset of patients, illustrating that route of delivery can alter effective tumor exposure (Brown et al., 2024). Trafficking- and matrix-directed engineering has improved intratumoral access in preclinical solid-tumor models (Caruana et al., 2015; Jin et al., 2019). These approaches are therefore most coherent when poor localization or stromal exclusion is the dominant limitation, rather than as generic additions to every CAR product.

A different intervention is required when exposure is adequate but recognition remains incomplete. Here, increasing persistence without increasing antigen coverage may simply prolong engagement with the fraction of tumor that is already recognizable. Multi-antigen and logic-gated designs instead aim to expand the effective antigenic space while controlling normal-tissue recognition. In glioblastoma models, synNotch-gated CAR circuits used one antigen to induce CAR expression against additional targets, improving control of heterogeneous tumors while retaining greater specificity than constitutive multi-target activation (Choe et al., 2021). The distinction between recognition rescue and persistence rescue is also apparent clinically. CARv3-TEAM-E cells combined EGFRvIII-directed CAR recognition with secretion of an EGFR-targeting T-cell-engaging antibody molecule to broaden activity against antigen-heterogeneous glioblastoma; the first three treated patients showed rapid radiographic regressions, yet recurrence occurred in two patients within a short interval (Choi et al., 2024). Thus, broader antigen coverage can improve early tumor control without necessarily solving the subsequent persistence problem.

Pressure-matched intervention can also target a dominant suppressive pathway once CAR T-cells reach the tumor. TGF-β provides a clinically instructive example. PSMA-directed CAR T-cells carrying a dominant-negative TGF-β receptor expanded in patients with metastatic castration-resistant prostate cancer and showed biological and transient antitumor activity, demonstrating that resistance to a defined suppressive pathway can be translated clinically; however, marked expansion was also accompanied by dose-dependent toxicity, emphasizing that removal of an external brake can expose other limits of the system (Narayan et al., 2022). Similar logic can be applied to defined metabolic or myeloid pressures, but such interventions should be prioritized when there is evidence that the corresponding pathway is functionally limiting. The purpose of this first intervention layer is therefore bottleneck relief: restore sufficient exposure, recognition or local permissiveness before asking an already constrained CAR T population to sustain stronger activation.

### 6.2 Preserve or expand a recoverable CAR T-cell pool

Once CAR T-cells are present within the tumor, the therapeutic question shifts from access to durability. Checkpoint modulation, cytokine support, metabolic reinforcement, and transcriptional engineering are most likely to preserve or expand CAR T-cells with residual proliferative capacity; terminally fate-restricted cells are expected to respond less effectively.

Checkpoint modulation illustrates why this distinction matters. Clinical experience with intrapleural mesothelin CAR T-cells followed by pembrolizumab established the feasibility of combining CAR T-cell therapy with PD-1 blockade in patients with malignant pleural disease (Adusumilli et al., 2021). However, the benefit of removing PD-1 signaling is not uniform across CAR configurations. In HER2 CAR-T cells, Andreu-Saumell et al. found that PD-1 disruption preferentially improved low-affinity CARs containing CD28 or ICOS costimulatory domains exposed to PD-L1, whereas high-affinity CARs and 4-1BB-containing constructs gained substantially less benefit (Andreu-Saumell et al., 2024). This is strong evidence against treating checkpoint blockade as a universal response to inhibitory-receptor expression: its value depends on how much the checkpoint is actually limiting an otherwise functional signaling system.

Cytokine support can similarly enlarge the persistent pool, but recent clinical evidence also defines its safety boundary. In patients with GPC3-positive solid cancers, IL-15-armored GPC3 CAR T-cells achieved substantially greater expansion than conventional GPC3 CAR T-cells and produced objective antitumor responses; tumor-infiltrating cells from responders also displayed distinct transcriptional features. Increased activity, however, was accompanied by a higher incidence of cytokine-release syndrome (CRS), requiring cytokine blockade or activation of an inducible safety switch in some patients (Steffin et al., 2025). Cytokine armoring should therefore be framed as a method for sustaining or expanding competent cells, not as an unrestricted means of overcoming terminal exhaustion. The same principle applies to metabolic support: restoring glucose utilization or mitochondrial capacity is most rational when viable CAR T-cells remain but their energetic reserve has become limiting, rather than when the dominant problem is irreversible loss of developmental plasticity.

Transcriptional support provides a more direct connection to the fate model developed above. c-Jun engineering established that manipulation of the AP-1 network can increase CAR T-cell resistance to chronic stimulation (Lynn et al., 2019), while newer work has moved this concept toward preservation of a defined regenerative compartment. In solid-tumor CAR T models, AP-1 modulation enabled formation of an intratumoral PD-1^+^TCF1^+^ stem-like reservoir that remained responsive to PD-1 blockade (Snyder et al., 2026). These findings suggest that effective reinvigoration requires a responsive precursor-like population that can expand after inhibitory signaling is relieved. An alternative is to reduce signaling rather than add stimulation. Weber et al. showed that temporary interruption of CAR signaling restored substantial function in exhausted CAR T-cells and induced transcriptional and epigenetic remodeling (Weber et al., 2021). Transient rest therefore introduces a distinct intervention principle: when chronic signaling itself is driving progressive dysfunction, periodically reducing receptor activity may preserve more function than continuously reinforcing activation. Together, checkpoint modulation, cytokine support, AP-1 engineering and controlled rest are best conceptualized as rescue strategies for a recoverable population. Their expected value should decline as that population is progressively replaced by fate-restricted cells.

### 6.3 Restart when CAR T-cell fate is deeply constrained

When CAR T-cells are poorly expandable, depleted, strongly manufacturing-imprinted or dominated by stable exhaustion-associated programs, continued rescue of the existing population may have diminishing returns. At this point, intervention moves upstream from reinvigoration toward rebuilding the cellular starting state.

Manufacturing provides the most practical entry point. Ghassemi et al. demonstrated that CAR T-cells can be generated within approximately 24 h without conventional prolonged activation and expansion; these minimally manipulated cells retained a less differentiated phenotype and greater per-cell antitumor potency than conventionally manufactured CAR T-cells (Ghassemi et al., 2022). Starting-cell selection provides a complementary approach. CAR T-cells manufactured from preselected naïve/stem-memory populations showed greater expansion and persistence and more durable antitumor activity than products generated from unselected T cells in experimental models (Arcangeli et al., 2022). These strategies do not attempt to reverse terminal exhaustion after it occurs; they reduce the probability that the next product enters the tumor with limited proliferative reserve.

Restarting may also require correcting the CAR itself. If dysfunction is partly imprinted by tonic signaling, high receptor density or inappropriate receptor biophysics, changing the T-cell starting population without correcting receptor design would reproduce the same liability. Chen et al. showed that positively charged patches in CAR antigen-binding domains promote receptor clustering and tonic signaling; rational charge-density tuning optimized tonic signaling and improved CAR T-cell fitness while preserving antigen recognition (Chen et al., 2023). Thus, CAR redesign belongs in this restart layer when the failure mechanism is intrinsic to receptor architecture rather than to the tumor alone.

Deeper transcriptional or epigenetic reprogramming is conceptually attractive but should be presented more cautiously than remanufacturing or receptor redesign. Gene-editing platforms such as CRISPR–Cas provide increasingly tractable approaches for modifying therapeutic T cells, including CAR T-cells, through targeted disruption or insertion of genes that regulate activation, persistence and inhibitory signaling (Qiu et al., 2022). Within the exhaustion context, DNMT3A deletion provides a more direct example: it preserved stem-like programs and improved CAR T-cell function during prolonged stimulation, demonstrating that manipulation of epigenetic regulation can alter exhaustion trajectory (Prinzing et al., 2021).

The practical distinction is that rescue works on the existing population, whereas restart attempts to create a new population with a higher tolerance for subsequent pressure. The deeper the loss of proliferative and epigenetic plasticity, the stronger the rationale for shifting from post-infusion reinvigoration toward product replacement, manufacturing optimization or receptor redesign.

### 6.4 Combination and sequencing strategies

Combination strategies should be selected according to the dominant tumor barrier and the functional state of the CAR T-cell population. Limited tumor access may favor trafficking- or stroma-directed approaches, whereas inadequate antigen coverage may require broader recognition. Once tumor exposure and recognition are sufficient, interventions that support CAR T-cell persistence or function become more relevant. This principle is consistent with the broader immunotherapy field, where rational combinations are increasingly guided by the underlying immune context and mechanisms of resistance rather than by empirical addition of agents (Li et al., 2026). Emerging checkpoints beyond PD-1/PD-L1 may further expand the range of potential partners for CAR T-cell therapy, although their value will require careful evaluation in the appropriate tumor and cellular context.

Spatially controlled engineering provides one way to coordinate combination signals while limiting systemic exposure. SynNotch circuits can restrict CAR expression to sites where a priming antigen is encountered and reduce tonic signaling associated with constitutive CAR expression (Choe et al., 2021). Endogenous regulatory programs have also been rewired to drive cytokine payload expression preferentially in tumor-engaged CAR T-cells (Chen et al., 2025). Similarly, CAR T-cells engineered to secrete a PD-L1-targeted IL-12 fusion localized immunomodulatory activity to PD-L1-positive tumors and reduced systemic inflammatory toxicity in preclinical models (Murad et al., 2026). Together, these approaches show that additional signals can be coupled to the tissue or activation context in which they are most likely to be useful.

Early clinical studies provide complementary examples of temporal sequencing. In H3K27M-mutant diffuse midline glioma, patients first received intravenous GD2-CAR T-cells, and those with clinical or radiographic benefit were eligible for subsequent intracerebroventricular infusions (Monje et al., 2025). This represents a response-informed transition from systemic to locoregional delivery. In malignant pleural mesothelioma, intrapleural mesothelin-directed CAR T-cell therapy was followed several weeks later by pembrolizumab (Adusumilli et al., 2021), providing a clinical precedent for temporally separating regional CAR T-cell therapy from checkpoint blockade.

These examples remain early-phase precedents rather than validated adaptive treatment algorithms. Future trials could incorporate predefined reassessment points after CAR T-cell infusion and use changes in tumor access, antigen expression, CAR T-cell expansion, and residual plasticity to guide subsequent intervention. Persistent antigen with poor CAR T-cell accumulation could favor a delivery- or trafficking-directed strategy, whereas emerging antigen loss could prompt broader targeting. A population that retains regenerative potential may remain amenable to functional rescue, while marked loss of proliferative capacity may favor product replacement or redesign. Prospective multi-stage studies will be needed to determine whether such pressure- and fate-matched sequencing improves outcomes over fixed combination regimens. Table 4 summarizes a stage-based framework for assessing CAR T-cell dysfunction and guiding subsequent intervention.

**Table 4:** Mechanism-informed assessment framework for solid-tumor CAR T-cell dysfunction.

Time point	Core question	Priority assays	Likely interpretation	Potential intervention direction	Avoid/caution
Pre-infusion	Is the target adequate and safe? Is the product already vulnerable?	Multi-region antigen IHC/IF; normal-tissue expression; product Tn/Tscm/Tcm; tonic signaling; serial stimulation	Antigen heterogeneity, narrow safety window, or manufacturing-imprinted vulnerability	Multi-antigen or logic-gated CAR; affinity tuning; optimized manufacturing	Avoid increasing CAR potency when the antigen safety window is narrow
Early post-infusion	Did CAR T-cells expand and reach the tumor?	Blood CAR kinetics; Ki-67; TCF1/CD127; imaging or biopsy when feasible	Expansion failure, exposure failure, or early suppression	Regional delivery; trafficking support; cytokine or metabolic support	Do not diagnose terminal exhaustion too early
Non-response	Are CAR T-cells absent, excluded, or suppressed?	Tumor biopsy; CAR localization; antigen retention; TGF-β, myeloid and stromal markers	Spatial exclusion or pressure-dominated suppression	Stromal targeting; myeloid targeting; dnTGFβR; metabolic support	Checkpoint-only rescue may fail if CAR T-cells are absent from tumor
Initial response followed by progression	Antigen escape or fate collapse?	Re-biopsy antigen; CAR persistence; Tpex/Tex balance; scRNA/scATAC if feasible	Antigen loss, reversible suppression, or terminal Tex	Multi-antigen redesign; checkpoint/TME relief if Tpex-like pool remains; remanufacturing or product replacement may be considered when strongly fate-restricted states predominate	PD-1-only rescue is weak if Tpex-like pool is lost
Late relapse	Is the product still recoverable?	TCF1/TCF7, TOX/NR4A, TCF7 methylation, ATAC/multiome, serial stimulation	Fate-dominated terminal dysfunction	Re-engineering; epigenetic reprogramming; Tn/Tscm-based remanufacturing	Avoid stacking stimulatory agents onto deeply fate-restricted cells

## 7 Conclusions

vSolid-tumor CAR T-cell failure arises from interacting antigenic, spatial, metabolic, immunoregulatory and product-intrinsic pressures. These pressures do not invariably produce a single dysfunctional state, but they can converge on exhaustion-associated fate trajectories that impair CAR T-cell expansion, serial killing and long-term persistence. Persistent or antigen-independent tonic CAR signaling can promote exhaustion-prone states in CAR T-cells (Long et al., 2015). Mechanistically, sustained NFAT activity without adequate AP-1 cooperation can induce exhaustion-associated transcriptional programs (Martinez et al., 2015), including NR4A- and TOX/TOX2-associated regulatory circuits that reinforce CAR T-cell dysfunction (Chen et al., 2019; Seo et al., 2019). Consistent with this model, insufficient AP-1 activity limits productive CAR T-cell function, whereas c-Jun reinforcement improves resistance to exhaustion (Lynn et al., 2019). These transcriptional changes are accompanied by dynamic chromatin remodeling and DNMT3A-dependent DNA methylation that progressively restrict memory-associated plasticity and stabilize dysfunctional states (Gennert et al., 2021; Prinzing et al., 2021). CAR T-cell populations may consequently redistribute along a continuum that includes TCF1-positive progenitor-like exhausted states with residual proliferative and regenerative capacity and more terminally dysfunctional states with limited reversibility (Jung et al., 2022; Zheng et al., 2021). Exhaustion should therefore be regarded as a major convergent fate trajectory in solid-tumor CAR T-cell failure, while recognizing that other non-exhaustion dysfunctional programs may also contribute and may differ according to CAR design and costimulatory architecture (Selli et al., 2023).

Several translational questions remain unresolved. A standardized CAR T-cell-specific definition of progenitor-like exhaustion has not yet been established, and the relationship between putative CAR T-cell Tpex-like states and canonical Tpex populations described in chronic infection or endogenous tumor-reactive T cells remains incompletely defined. The temporal boundary between reversible suppression and stable fate restriction is also unknown, making it difficult to determine when checkpoint modulation, cytokine support or metabolic rescue can restore durable activity and when product redesign or remanufacturing is required. Clinically accessible measurements of infusion-product composition and peripheral CAR T-cell expansion and persistence provide important information (Kaczanowska et al., 2024; Ploch et al., 2024; Zduniak et al., 2025), but paired blood–tumor studies will be required to establish whether these measurements reflect intratumoral antigen geography, spatial exclusion, suppressive cellular neighborhoods and epigenetic restriction. Longitudinal analysis of infusion products, peripheral blood and tumor samples will therefore be necessary to develop and validate composite biomarkers of residual CAR T-cell plasticity. The safety of exhaustion-resistance engineering must also be evaluated in a regulator-specific manner. NR4A inhibition, AP-1 reinforcement and modulation of DNA methylation may improve CAR T-cell activity in preclinical models, whereas evidence from chronic-infection models indicates that TOX can also support T-cell persistence and adaptation during prolonged antigen exposure (Yao et al., 2019). Whether this adaptive function applies to CAR T-cells remains uncertain. The effects of manipulating exhaustion-associated pathways are therefore likely to depend on the selected target, intervention timing and magnitude, CAR architecture, antigen burden and tissue context.

Future development should prospectively evaluate an investigational, diagnosis-guided framework in which the dominant tumor pressure and the residual plasticity of the CAR T-cell population are assessed together. Tumors characterized by inadequate CAR T-cell exposure may warrant trafficking enhancement, regional delivery, vascular modification or stromal remodeling. Antigen-heterogeneous tumors may require multi-antigen, tandem or logic-gated recognition, whereas CAR T-cell populations that retain progenitor-like features may be more amenable to checkpoint modulation, cytokine support or relief of microenvironmental pressure. In contrast, deeply differentiated and epigenetically restricted populations may require CAR redesign, transcriptional or epigenetic reprogramming, selection of less differentiated starting cells or remanufacturing, although these approaches require prospective validation. Emerging in vivo CAR T-cell strategies may avoid prolonged ex vivo expansion and therefore have the potential to reduce manufacturing-associated differentiation, but this proposed advantage and its effects on exhaustion, tissue specificity, CAR expression, persistence and safety remain to be experimentally and clinically established (Bui et al., 2024). Progress will ultimately depend on determining whether pressure- and state-matched interventions can preserve CAR T-cell infiltration, expansion, antitumor function and persistence more effectively than empirically selected combinations.

## CRediT authorship contribution statement

**Pei Wang:** Writing – review & editing, Writing – original draft, Visualization, Project administration, Investigation, Conceptualization. **Yuehong Zhou:** Writing – review & editing, Visualization, Investigation, Data curation. **Yuan Wang:** Supervision. **Yangyang Li:** Supervision. **Xue Xiao:** Supervision. **Chenhui Wang:** Supervision, Project administration, Conceptualization.

## Declaration of competing interest

The authors declare that they have no known competing financial interests or personal relationships that could have appeared to influence the work reported in this paper.
